# Cyclodextrin-mediated enhancement of gastrointestinal drug delivery: unveiling mucoadhesive and mucopenetrating synergy

**DOI:** 10.1007/s13346-025-01832-w

**Published:** 2025-03-20

**Authors:** Soheil Haddadzadegan, Ahmad Saleh, Florina Veider, Patrick Knoll, Flavia Laffleur, Gergely Kali, Andreas Bernkop-Schnürch

**Affiliations:** 1 ThioMatrix Forschungs- und Beratungs GmbH, Trientlgasse 65, Innsbruck, 6020 Austria; 2 Center for Sustainable Materials (SusMat), School of Materials Science and Engineering, Nanyang Technological University, Singapore, 639798 Singapore; 3 Department of Pharmacy, Universitas Mandala Waluya, Kendari, Southeast Sulawesi 93231 Republic of Indonesia; 4 Center for Chemistry and Biomedicine, Department of Pharmaceutical Technology, Institute of Pharmacy, University of Innsbruck, Innrain 80/82, Innsbruck, 6020 Austria

**Keywords:** Drug delivery, Mucoadhesive, Thiomer, S-protection, Cyclodextrin, In vivo

## Abstract

This study evaluates the in vivo mucoadhesive properties of thiolated cyclodextrins (CDs) with varying S-protection using polyethylene glycol (PEG) of different chain lengths. Free thiol groups of thiolated β-CDs (CD-SH) were S-protected with 1 kDa and 2 kDa PEG bearing a terminal thiol group, leading to third-generation of thiolated CDs (CD-SS-PEG). The structure of these thiolated CDs was confirmed and characterized by FT-IR, 1 H NMR, and colorimetric assays. Thiolated and S-protected CDs were evaluated regarding viscosity, cellular uptake and, in vitro and in vivo mucoadhesion. The viscosity of CD-SH, CD-SS-PEG 1 kDa, and CD-SS-PEG 2 kDa mixtures with mucus increased 9-, 7-, and 5.5-fold, respectively, compared to unmodified CD within 3 h. Cellular uptake on Caco-2 cells was 1.75 times higher for highly thiolated CDs than for unmodified CD. In vitro residence time on porcine intestine was prolonged 7-, 8.4-, and 7.9-fold for CD-SH, CD-SS-PEG 1 kDa, and CD-SS-PEG 2 kDa, respectively. In vivo results indicated CD-SS-PEG 1 kDa had the highest potential. Our comprehensive in vitro, ex vivo, and in vivo ffindings demonstrate that CD-SS-PEG 1 kDa is a highly promising candidate for mucoadhesive drug delivery systems.

**Graphical Abstract:**

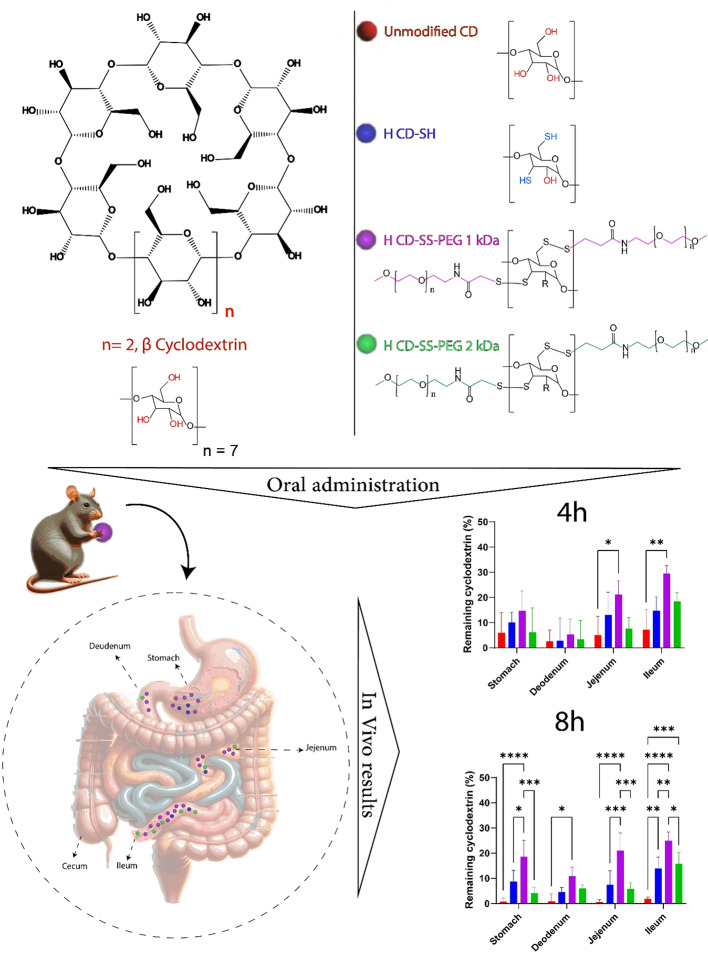

**Supplementary Information:**

The online version contains supplementary material available at 10.1007/s13346-025-01832-w.

## Introduction

Cyclodextrins (CDs) and their derivatives serve as effective excipients with the potential to enhance the bioavailability of a wide array of active pharmaceutical ingredients (APIs) [1]. Due to their ability to enhance drug solubility, CDs are used in a variety of pharmaceutical products [2]. Because of their relatively less hydrophilic central cavity, CDs allow for the incorporation of lipophilic drugs, resulting in enhanced aqueous solubility, improved chemical stability, and masking undesirable tastes or odors associated with the APIs [3]. Because of their benefits in drug delivery, several cyclodextrins (CDs) and their derivatives have been officially recognized as pharmaceutical excipients. These include α-CD, β-CD, γ-CD, hydroxypropyl β-CD (HP-β-CD), hydroxypropyl γ-CD (HP-γ-CD), randomly methylated β-CD (RM-β-CD), and sulfobutylated β-CD (SB-β-CD) [2]. Nevertheless, their limited mucoadhesive characteristics constrain their suitability for administration through this pathway, given the relatively brief duration of contact with mucosal membranes [4]. To overcome this shortcoming, mucoadhesive thiolated CDs were introduced [5]. Evidence for their potential has been provided by numerous studies in recent years [4, 6–9]. These carriers can form disulfide bonds with cysteine-rich subdomains of mucus glycoproteins, resulting in robust mucoadhesive properties [10]. As the crucial factor for mucoadhesion is the degree of thiolation, the more hydroxyl groups are substituted by thiol groups, the stronger the mucoadhesion [11]. The main limitation of thiolated CDs is that the free thiol groups are prone to oxidation, making them susceptible to oxidation to disulfide bonds, decreasing the number of free thiol groups [12]. Furthermore, the thiol groups of these CDs readily interact with cysteines within the outer, loosely bound mucus gel layer, which is rapidly eliminated by the mucus turnover process. This underscores the clear requirement for enhanced interactions of thiolated CDs. Second and third generations of thiolated CDs, named as S-protected ones, received more interest recently [11, 13]. Free thiol groups can be partially and reversibly deactivated using various S-protecting agents. Depending on the reactivity of these agents, 2nd and 3rd generation thiomers can be synthesized. The second generation is produced by highly reactive sulfhydryl ligands, for example, 2-mercaptonicotinic acid (MNA). In contrast, in case of the 3rd generation, sulfhydryl ligands with lower reactive, such as cysteine or glutathione, are used for S-protection [14]. Because of their low reactivity 3rd generation thiolated CDs can freely diffuse into deeper mucus regions as they do not immediately form new disulfide bonds with mucus glycoproteins [11], resulting in higher mucoadhesion [15]. To date, third-generation thiolated CDs have been subjected to in vitro assessments of their mucoadhesive properties, with no corresponding in vivo evaluations.

Hence, the objective of this study was to synthesize first and third-generation thiolated CDs featuring different degrees of thiolation (low and high), as well as distinct densities and lengths of S-protecting PEG chains. This synthesis aimed to comprehensively compare their mucoadhesive characteristics through both in vitro and in vivo assessments. Unprotected thiolated β-CD (1st generation) was synthesized by substituting hydroxyl groups with thiol moieties on the glucose subunits via direct conversion with phosphorous pentasulfide for high thiolations. In the case of third generation thiolated CDs, thiol-terminated PEG chains of 1 and 2 kDa were employed to provide S-protection for the thiolated CDs. We evaluated how varying degrees of thiolation and distinct types of S-protection affect in vitro cell viability. Furthermore, the assessment involved membrane toxicity, cellular uptake, and mucoadhesion. Conclusively, the in vivo mucoadhesive characteristics of thiolated cyclodextrins loaded with the model drug Coumarin 6 (C_6_) were assessed using a rat model.

## Experimental

### Materials

Beta CD (β-CD, catalogue code: CY-2001; α-1,4 glycosidic bond connected cyclic anhydroglucose; fine chemical grade; purity > 95%; molecular weight: 1135.0 g/mol) and thiolated β-CD (catalogue code: CY-2224; heptakis (6-deoxy-6-thio)-beta-cyclodextrin; purity > 97%; molecular weight: 1247.4 g/mol) were obtained from CycloLab Ltd. 3-(2-Pyridyldithio) propionic acid methoxy polyethylene glycol (mPEG-OPSS) with PEG chains of 1 kDa and 2 kDa were sourced from Abbexa. Chemicals such as thiourea (CS(NH2)2, ≥ 99%), 2-mercaptonicotinic acid (2-MNA, ≥ 98%), and 5,5′-dithiobis(2-nitrobenzoic acid) (Ellman’s reagent, ≥ 98%) were supplied by Sigma-Aldrich, Vienna, Austria. Additional materials from Sigma-Aldrich included Spectra/PorÒ dialysis membranes with molecular weight cut-offs (MWCO) of 1000 and 3500 Da, Hanks’ balanced salt solution (HBSS), resazurin sodium salt (7-hydroxy-3 H-phenoxazin-3-one 10-oxide, dye content ≥ 75%), cysteine (≥ 98%), sodium borohydride (NaBH4, ≥ 99%), dimethyl sulfoxide-d6 (DMSO-d6, ≥ 99.9%), Coumarin 6 (C6), minimum essential eagle medium (MEM), sodium chloride (NaCl), methanol (≥ 95–99%), phosphorus pentasulfide (P4S10, 99%), tetramethylene sulfone (sulfolane, 99%), and Triton-X 100. The cell culture medium was prepared in line with published protocols. Fetal bovine serum (FBS) and 100 mM phosphate-buffered saline (PBS) at pH 6.8 were obtained from Gibco, a part of Invitrogen, Lofer, Austria. All other reagents used were of analytical grade and sourced from commercial suppliers.

### Synthesis of first-generation of thiolated CD

#### Synthesis of highly thiolated cyclodextrin (h CD-SH)

Highly thiolated CDs (h CD-SH) were synthesized according to a previously described method [2]. In brief, a mixture of β-CD (0.5 g, 0.44 mmol) and phosphorus pentasulfide (2.4 g, 5.3 mmol) was prepared in 15 mL of sulfolane at 30 °C. Triethylamine (1 mL, 13.5 mmol) was then introduced into the reaction. The solution was gradually heated to 130 °C and left stirring overnight. Afterward, the temperature was reduced to 80 °C, followed by the gradual addition of water. The resulting h CD-SH precipitate formed in the hot water, was filtered, rinsed with hot water, and dried to achieve a constant weight. The yield for highly thiolated cyclodextrins was observed to be 85%. In this research, heptakis(6-deoxy-6-thio)-β-CD was referred to as low thiolated CD (l CD-SH). Blue illustrations are used to represent the first generation of thiolated CDs in this study.

### Synthesis of third generation of thiolated CD

#### Synthesis of PEG-protected CDs (CD-SS-PEG 1 kDa)

To develop 3rd generation thiolated CDs (CD-SS-PEG), the thiolated CDs were reacted with methoxy PEGylated 3-(2-pyridyldithio) propionic acid (mPEG-OPPSS 1 kDa). For this purpose, 50 mg of the first-generation thiolated CDs were dissolved in demineralized water, followed by the addition of increasing amounts of mPEG-OPPSS. Specifically, 150 mg (0.11 mmol) of mPEG-OPPSS 1 kDa was added to the low thiolated CD (l CD-SH) solution, and 400 mg (0.27 mmol) of mPEG-OPPSS 1 kDa was added to the high thiolated CD (h CD-SH) solution. The reaction mixture was stirred for 24 h and then dialyzed in the dark for three days using a Spectra/PorÒ dialysis membrane (MWCO: 3500 Da). After dialysis, the product was freeze-dried. The purity of the resulting CD-SS-PEG was confirmed through thin-layer chromatography, utilizing silica as the stationary phase and a chloroform/methanol mixture (85/15%) as the mobile phase. Detection was carried out by exposing the chromatographic plates to iodine vapor. PEGylation provided protection for the low thiolated CD (l CD-SS-PEG 1 kDa) and the high thiolated CD (h CD-SS-PEG 1 kDa). The yield for l CD-SS-PEG 1 kDa and h CD-SS-PEG 1 kDa was observed to be 29.8% and 37%, respectively. Throughout the study, purple illustrations, including those in the graphical abstract, were used to represent CD-SS-PEG 1 kDa.

#### Synthesis of PEG-protected cds (CD-SS-PEG 2 kDa)

**Fig. 1:**
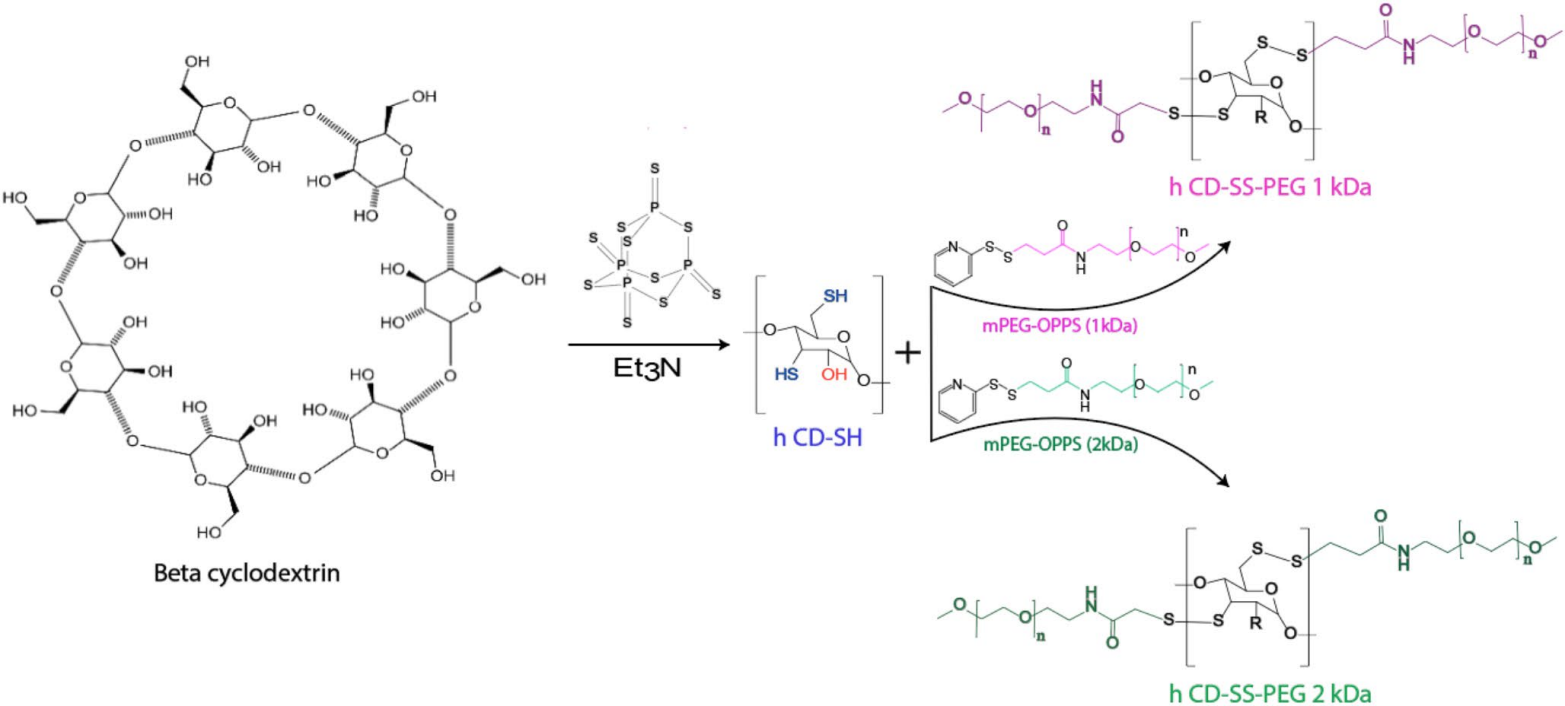
Synthetic pathways for hCD-SH, hCD-SS-PEG (1 kDa), and hCD-SS-PEG (2 kDa). The hydroxyl groups of native CD were directly converted into thiol groups. The formation of hCD-SS-PEG (1 kDa) and hCD-SS-PEG (2 kDa) was achieved through disulfide bond formation between the thiolated CD and mPEG-OPSS (1 kDa and 2 kDa, respectively)

To produce 3rd generation thiolated CDs (CD-SS-PEG 2 kDa), the thiolated CDs were reacted with methoxy PEGylated 3-(2-pyridyldithio) propionic acid (mPEG-OPPSS). For this synthesis, 50 mg of the first-generation thiolated CDs were dissolved in demineralized water, and varying amounts of mPEG-OPPSS were incrementally added. Specifically, 250 mg (0.11 mmol) of mPEG-OPPSS 2 kDa was added to the low thiolated CD (l CD-SH) solution, while 600 mg (0.27 mmol) of mPEG-OPPSS 2 kDa was added to the high thiolated CD (h CD-SH) solution. The mixture was stirred for 24 h, followed by a three-day dialysis in the dark using a Spectra/PorÒ dialysis membrane (MWCO: 3500 Da). The remaining steps of this procedure were carried out similarly to those described in the “Synthesis of PEG-protected CDs (CD-SS-PEG 1 kDa)” section. As a result of PEGylation, protection was achieved for both low thiolated CD (l CD-SS-PEG 2 kDa) and high thiolated CD (h CD-SS-PEG 2 kDa). The yield for l CD-SS-PEG 1 kDa and h CD-SS-PEG 1 kDa was observed to be 41% and 45.3%, respectively. Green illustrations, including those in the graphical abstract, were used to represent CD-SS-PEG 2 kDa throughout this study. The chemical reactions are depicted in Fig. 1.

### Characterization of thiolated and S-protected CD by FTIR spectroscopy and 1 H NMR

Fourier transform infrared (FT-IR) spectra of both thiolated and S-protected CDs were acquired using a Bruker ALPHA FTIR system (Billerica, MA, USA) equipped with a platinum attenuated total reflection (ATR) module. The powdered samples were directly placed on the ATR crystal for analysis. Spectral data were collected over a range of 4000 cm⁻¹ to 500 cm⁻¹, with a resolution of 4 cm⁻¹ and 32 scans per sample. The final FT-IR spectra represented the average of these 32 scans. For nuclear magnetic resonance (NMR) measurements, all analyses were conducted using a Bruker “Mars” 400 MHz Avance 4 Neo spectrometer (Billerica, MA, USA) in DMSO-d6. Approximately 5 mg of each dry sample was dissolved in 0.7 mL of the deuterated solvent. Chemical shifts were recorded in parts per million (ppm), with the DMSO-d6 solvent peak serving as the internal standard at δ 2.49 ppm.

### Thiol content

The free thiol content on the backbone of thiolated CDs and S-protected thiolated CDs was assessed using Ellman’s reagent, following a method previously established by our research group, which involved a standard curve generated with varying cysteine concentrations [5]. In brief, a photometric assay was performed at a wavelength of 450 nm with Ellman’s reagent (DTNB) to measure the levels of free thiols and disulfides, both before and after reduction with NaBH4. Free thiol groups were quantified in a 0.5 M phosphate buffer at pH 8.0. For disulfide quantification, the samples were reduced with sodium borohydride in 8.3 mM Tris buffer at pH 7.6, using a final NaBH4 concentration of 2.8% (w/v).

### Safety screening

#### Resazurin assay

The cytotoxicity of CDs at a concentration of 0.25% (w/v) was assessed on four distinct cell lines: Caco- 2 (human caucasian adenocarcinoma cell line, ECACC: 86010202), HEK (human embryonic kidney cells, ECACC: 85120602), HT-29 (human colorectal adenocarcinoma, ECACC: 12040401), and HeLa (human cervical carcinoma, ECACC: 93021013). A total of 2.5 × 10⁴ cells per well were plated in a 24-well plate and maintained for 10 days in Earle’s balanced salts, supplemented with 2.0 mM L-glutamine, 10% FBS, and 1% penicillin-streptomycin, under a 5% CO₂ atmosphere at 37 °C. The culture medium was refreshed every other day. Cells exposed to 1% (w/v) Triton X-100 served as the positive control, whereas a buffer composed of 25 mM HEPES and 268 mM glucose at pH 7.4 was used as the negative control. Test samples were formulated in glucose HEPES buffer at a final concentration of 0.25% (w/v). Prior to testing, cells were washed twice with 100 µL of phosphate-buffered saline (PBS), followed by the addition of 500 µL of test solutions to the wells. The plates were then incubated at 37 °C in a 5% CO₂ environment for 24 h. After incubation, the test solutions were removed, and cells were washed twice with PBS. Subsequently, 500 µL of a 2.2 mM resazurin solution was added to each well, and cells were incubated for an additional 3 h under the same conditions. Fluorescence intensity was determined by collecting 100 µL aliquots from each well and measuring with a microplate reader at excitation and emission wavelengths of 540 nm and 590 nm, respectively. Cell viability was then calculated using the following equation:

$$Cell{\rm{ }}\,viability{\rm{ }}\left( \% \right){\rm{ }} = {\eqalign{& {\rm{intensity\,of\,sample}} \cr& {\rm{}} - {\rm{intensity\,of\,negative\,control}} \cr} \over \eqalign{& {\rm{intensity\,of\,positive\,control}} \cr& - {\rm{intensity\,of\,negative\,control}} \cr} }{\rm{*}}10$$

#### In vitro hemocompatibility assay

The impact of various types of CDs on the integrity of human erythrocyte membranes was assessed using an in vitro hemolysis assay with human erythrocyte concentrate, generously donated by Tirol Kliniken GmbH, Innsbruck, Austria [16]. For the experiments, erythrocyte concentrate was used without undergoing filtration or centrifugation but was instead diluted with a buffer before testing. A 1:100 (v/v) dilution was prepared in glucose-HEPES buffer (268 mM glucose and 25 mM HEPES, pH 7.4). To verify the structural integrity of red blood cells (RBCs) before analysis, the diluted suspension was centrifuged at 8000 rpm for 10 min using a MiniSpin Centrifuge (Eppendorf, Hamburg, Germany). Test solutions containing 0.25% (w/v) of both thiolated and unmodified CDs, formulated in the same HEPES buffer, were mixed with the RBC suspension in equal volumes. The samples were incubated at 37 °C with continuous shaking at 200 rpm in an orbital shaker (Orbital Shaker – Incubator ES-80, Grant Instruments Ltd., Cambridge, England) for various durations (1, 4, 8, and 24 h). After incubation, another centrifugation step was performed at 8000 rpm for 10 min using the same centrifuge, and the supernatants were examined at 415 nm using a UV-spectrophotometer (Tecan Infinite M200; Grödig, Austria). A glucose-HEPES buffer served as the negative control, while the positive control consisted of a 1% (w/v) Triton X-100 solution in the same buffer. The percentage of hemolysis was then calculated using the following equation:

$${\rm{Hemolysis\% }} = {\rm{}}{\eqalign{& {\rm{Absorbance\,of\,sample}} \cr& - {\rm{Absorbance\,of\,negative\,control}} \cr} \over \eqalign{& {\rm{Absorbance\,of\,positive\,control}} \cr& - {\rm{Absorbance\,of\,negative\,control}} \cr} }{\rm{*}}100$$

### Purification of mucus

Porcine small intestines were obtained from a local slaughterhouse. A 10 to 20 cm segment was collected from freshly slaughtered pigs and then longitudinally dissected. Any parts containing chyme were removed and discarded. The mucus was carefully scraped off the mucosa using the tip of a finger. The collected raw mucus was placed into a falcon tube and diluted with a 0.1 M aqueous NaCl solution at a ratio of 2 g of mucus to 10 mL of NaCl. This mixture was gently stirred on ice for one hour, followed by centrifugation at 10,500 rpm for another hour. The supernatant, along with any blood and granular yellowish-dark material adhering to the sides and bottom of the tube, was discarded. The remaining mucus was resuspended in a 0.1 M NaCl solution and gently stirred on ice for one hour, using half the volume of NaCl as in the initial step, then subjected to the same centrifugation process. After the final centrifugation, the supernatant was removed, and the purified mucus was homogenized for further use.

### Rheological studies

A cone-plate rheometer (HAAKE Mars Rheometer, 40/60, Thermo Electron GmbH, Karlsruhe, Germany, Rotor: C35 /1, D = 35 mm) was used to perform rheological measurements, maintaining the temperature at 37 °C with a 0.052 mm gap between the plate and cone. Samples of unmodified, thiolated, and PEG-modified CDs were prepared in 0.1 M phosphate buffer (pH 6.8) and combined with purified mucus at a 1:5 mass ratio. The mixtures were incubated at 37 °C for 3 h. To determine the linear viscoelastic region, oscillatory strain sweep tests were conducted at 37 °C with a 1 Hz frequency and a strain range of 0.01–50 Pa. A sample containing only unmodified CD and mucus was used as a reference. Before measurements, samples were placed on the lower plate and allowed to stabilize for 60 s. Rheological characteristics were then analyzed at a shear stress of 0.1 Pa across a frequency spectrum of 0.1–20 Hz. The dynamic viscosity as a function of frequency was recorded using HAAKE Rheo Win 3 software.

### Labeling with C6

To assess mucoadhesive properties, unmodified and thiolated CDs were fluorescently labeled using host-guest complexation with C6. Specifically, 50 mg of both modified and unmodified CDs were hydrated in 50 mL of demineralized water, with the pH of the solutions adjusted to 6.5 using 100 mM HCl. Subsequently, 1 mL of a 0.02% (m/v) C6 solution in 96% ethanol was added to each CD solution and stirred at room temperature. After a 24-hour incubation period, the suspensions were filtered to remove any unbound dye, and the resulting filtrates were freeze-dried. The resulting complexes were then subjected to fluorescence analysis to evaluate their properties.

### In vitro evaluation of mucoadhesive properties

To evaluate mucoadhesive properties, porcine intestinal mucosa was freshly collected from a local slaughterhouse. Sections of the small intestine (4 × 2 cm) were prepared and attached to modified 50 mL Falcon tubes, positioned at an 80° angle inside an incubation chamber set at 37 °C with 100% humidity. The tissue surfaces were rinsed continuously for 10 min with 0.1 M phosphate buffer (pH 6.8) at a flow rate of 0.5 mL/min. Afterward, 5 mg of C6-labeled CD samples were applied separately to each mucosal section. Following a 20-minute incubation, buffer flow was resumed. Samples (5 mL) of the buffer that had passed over the mucosal tissue were collected at various time points (10, 20, 30, 40, 50, and 60 min). For reference, control samples containing 100% CD were prepared by dissolving 5 mg of CD in 5 mL of collected buffer after washing the mucus. Each sample was then combined with 1 mL of ethanol, incubated at 37 °C with shaking for one hour in the dark, and centrifuged at 13,400 rpm for 10 min at room temperature. A 100 µL aliquot from each supernatant was analyzed using a microplate reader (M200 spectrometer; Tecan Infinite, Grödig, Austria) to measure fluorescence intensity, with excitation and emission wavelengths set at 445 nm and 510 nm, respectively. All tests were conducted in triplicate.

### Cellular uptake studies

Cellular uptake was examined using Caco2 and HEK cell lines. Cells were seeded at a density of 5 × 10⁴ cells/mL and cultured for 11 days. After removing the MEM, the cells were incubated with CDs at a concentration of 0.05% (v/v). The experiment was conducted at both 37 °C and 4 °C. Following the incubation, the formulations were removed by washing the cells with ice-cold PBS, and 150 µL of trypsin was added for 5 min. To prevent trypsin-induced cellular damage, 500 µL of MEM was added to reduce its activity. The cells were then singularized by pipetting for 30 s. Cells from two wells were pooled into tubes to achieve adequate cell density, centrifuged at 800 rpm for 4 min, and washed twice with PBS to thoroughly remove any residual trypsin and MEM. Afterward, the cells were resuspended in PBS and passed through a cell strainer with a 70 μm pore size. The fluorescence signal of more than 10,000 cells was measured to determine the uptake of C6-labeled CDs, with consistent gating settings applied. Data were analyzed using FlowJo™ v10.8 software.

### In vivo studies

#### Animal research information

The study involving thiolated cyclodextrins was conducted on 16 male Sprague–Dawley rats, sourced from Janvier Labs (Saint Berthevin, France), with body weights ranging from 250 to 350 g. The authors have adhered to the ARRIVE guidelines. All experiments were performed in accordance with the Regulations of the Experimental Animal Administration, followed the Principles of Laboratory Animal Care and received approval from the Animal Ethical Committee in Vienna, Austria (Reference code: 2021 − 0.721.001). The in-vivo studies followed the EU Directive 2010/63 for the protection of animals used for scientific purposes guidelines. The rats were housed in a controlled-environment facility, maintaining stable temperature and humidity levels, with unlimited access to food and water. Rats were chosen as the model animal for this study due to their well-established use in biomedical research, offering a robust and reproducible system for studying physiological and pharmacological processes relevant to humans. Their genetic, biological, and behavioral characteristics closely mimic those of humans, making them suitable for investigating complex biological mechanisms. Additionally, their size facilitates ease of handling and sample collection, while their relatively short reproductive and life cycles allow for efficient observation of study outcomes. The extensive background knowledge available for Sprague–Dawley rats further supports their use, enabling comparison with existing literature and ensuring reliability in interpreting results. To minimize suffering, all procedures involving rats were carried out in strict adherence to ethical guidelines and under the supervision of trained personnel. At the end of the study, the rats were humanely sacrificed using CO₂ inhalation, a method recognized as a humane and effective means of euthanasia by the American Veterinary Medical Association (AVMA) Guidelines. This approach ensured minimal pain and distress to the animals while maintaining the integrity of the experimental outcomes.

#### Other statement

For the in vivo investigations, test samples were formulated at a concentration of 25 mg/mL. This concentration was established through preliminary tests. The dye’s amount needed to enable quantification was considered while ensuring it wouldn’t excessively obstruct the feeding needle. The rat subjects were stratified into four distinct cohorts, with each cohort encompassing four individuals. The initial group underwent oral administration of 500 µL of a solution containing 2.5% (m/V) unmodified CDs. In contrast, the second group was administered 500 µL of a solution comprising 2.5% (m/V) of a complex involving h CD-SH. The third group received h CD- SS-PEG 1 kDa, and the fourth group was subjected to h CD-SS-PEG 2 kDa. The animals underwent a 12-hour fasting period before the oral administration and were allowed unrestricted access to water throughout the duration of the experiment. At the 4-hour and 8-hour, four rats from each cohort were euthanized. Subsequently, the stomach and intestine were excised and subjected to extraction using 96% ethanol. The concentration of the solubilized dye was determined through fluorescence measurements, employing a calibration curve based on C_6_ dissolved in ethanol.

### Statistical data analysis

Statistical analyses were performed using the Student’s t-test, applying a confidence level of *p* < 0.05, utilizing GraphPad Prism (GraphPad Prism^®^, version 9, GraphPad Software, Inc.). Differences between multiple groups were assessed using one-way analysis of variance (ANOVA) with a 95% confidence interval (*p* < 0.05). For post-hoc analysis, Bonferroni’s test was employed to evaluate multiple comparisons. The data are presented as means ± SD, based on experiments conducted in triplicate.

## Results

### Synthesis and characterization of H CD-SH

Two distinct generations of thiolated CDs were synthesized, each with varying degrees of thiolation. The quantities of immobilized thiol groups and disulfide bonds produced during the reactions were quantified using Ellman’s assay and disulfide testing, with the results presented in Table 1. The successful formation of thiol groups on the CDs was verified through FT-IR and ¹H NMR spectroscopy. In the FT-IR spectra, a broad and weak peak between 2550 and 2700 cm⁻¹ (Figure S1, Supporting Information) corresponds to the R-S-H stretching vibrations, which consistently appeared following the thiolation process. The ¹H NMR spectrum of the thiolated product (Figure S2 in Supporting Information), recorded in DMSO-d6, indicated a decrease in the signals of hydroxyl groups at positions 2 and 3 (5.90 ppm) and position 6 (4.20 ppm). Additionally, new peaks emerged at 2.05 and 1.15 ppm, corresponding to the sulfhydryl groups.

### Synthesis and characterization of S-protected thiolated CD (CD-SS-PEG)

Thiolated CDs were S-protected using mPEG-OPSS with molecular weights of 1 kDa and 2 kDa. The successful S-protection was verified by FT-IR and ¹H NMR spectroscopy. The absence of the R-S-H stretching vibration in the IR spectra suggests that a thiol-disulfide exchange occurred. Additionally, the significant reduction in the intensity of thiol peaks in the ¹H NMR spectra, along with the appearance of PEG-related peaks at 3.5 ppm, further confirms the successful conjugation of PEG to the CDs.

**Table 1:** Chemical characterization of S-protected thiolated cds. Indicated values are means ± standard deviation of at least three experiments

	l CD-SH	h CD-SH	l CD-SS-PEG 1 kDa	l CD-SS-PEG 2 kDa	h CD-SS-PEG 1 kDa	h CD-SS-PEG 2 kDa
Free thiols [µmol/g]	3241 ± 98	6168 ± 42	12 ± 5	244 ± 17	69 ± 11	127 ± 7
Disulfides [µmol/g]	146 ± 11	587 ± 26	2696 ± 29	2188 ± 34	4172 ± 160	3690 ± 534

### Biological studies

#### Cytotoxicity

**Fig. 2:**
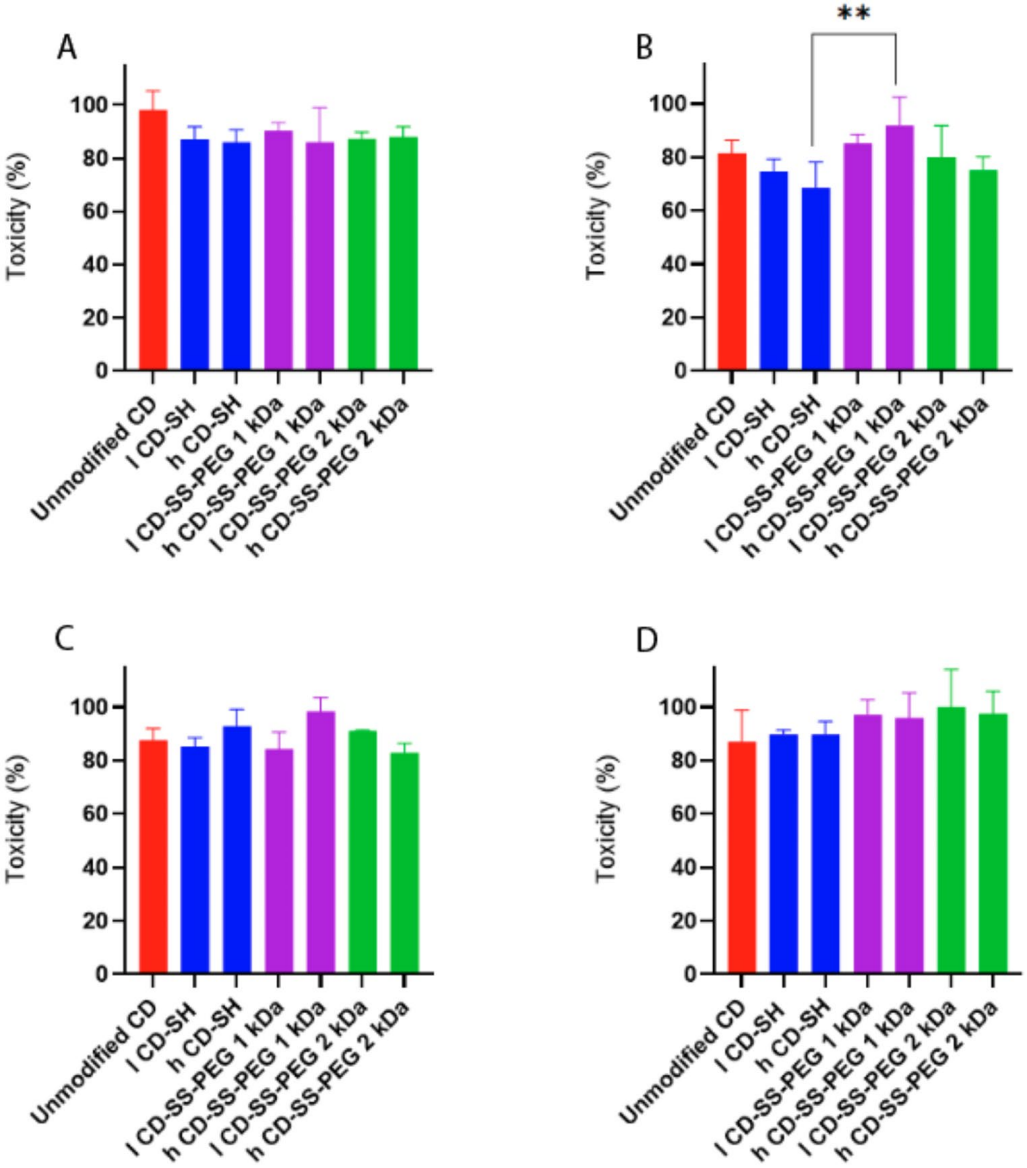
Viability of Caco-2 cells (**A**), HEK293 (**B**), Hela (**C**), and HT-29 (**D**) after treatment with unmodified and thiolated CDs at a concentration of 0.25% (w/v) after 4 h of incubation. Indicated values are means ± standard deviation of at least three experiments

Cytotoxicity is one of the essential features of a potential new excipient. The results of cytotoxicity studies of unmodified CD, CD-SH, CD-SS-PEG 1 kDa, and CD-SS-PEG 2 kDa on Caco-2, HEK 293, Hella, and HT-29 cells are shown in Fig. 2. Native CD and all thiolated, as well as S-protected thiolated CDs, did not show any significant cytotoxic effect during 4 h of incubation (p˂0.05) at applied concentration (viability higher than 80% for all the cell lines). This pattern was similar for the HT-29 cell line as well. In contrast, in case of the HEK 293 cell line, the viability of CD-SH for both low and high thiolated ones decreased below 80%. Interestingly, after S-protection, the viability of both l CD-SH and h CD-SH was increased. This improvement in toxicity was more obvious by S-protection with 1 kDa PEG.

#### Hemolysis assay

**Fig. 3:**
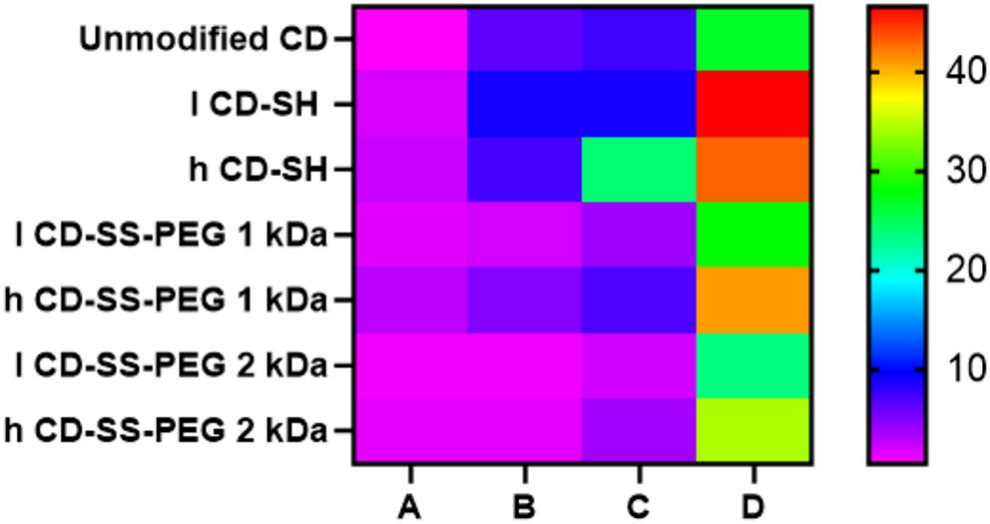
Hemolytic effect of 0.25% unmodified, thiolated, and S-protected thiolated CD in physiological glucose-HEPES buffer (268 mM glucose and 25mM HEPES pH 7.4) at time points of 1 h, 4 h, 8 h, and 24 h. Indicated values are means ± standard deviation of at least three experiments

The cell membrane of red blood cells (RBCs) is highly sensitive to environmental impacts [17]. A hemolysis assay is a valuable tool for assessing the potential membrane toxicity of CDs by measuring the degree of red blood cell (RBC) membrane disruption and subsequent hemoglobin release into the surrounding medium. This release can be quantified to determine toxicity. In this study, native, thiolated, and S-protected CDs were tested for their ability to induce hemoglobin release from fresh human erythrocytes using a hemolytic assay. According to ASTM (American Society for Testing and Materials) standards (E2524-08), a hemolysis rate above 5% is indicative of toxicity to RBCs. As illustrated in Fig. 3, neither unmodified nor thiolated CDs resulted in significant hemolysis after 1 h at a concentration of 0.25% (w/v). This finding aligns with a recent study by Veider et al., which also demonstrated minimal hemolysis with thiolated CDs, even at a higher concentration of 0.3% [9]. A higher degree of thiolation in CDs generally led to an increased percentage of hemolysis. For CDs with high levels of thiolation, hemolysis rose to 10% after 4 h. However, S-protection with PEG significantly reduced hemolysis to below 10%. Interestingly, for PEG-protected CDs, particularly l CD-SS-PEG, hemolysis remained under 5% even after 8 h, staying within the acceptable safety threshold. These findings suggest that the enhanced membrane interactions observed with CD-SH, compared to unmodified CD, may improve the potential of β-CD for endosomal escape due to thiolation.

### Rheological measurement

#### Viscosity measurement

**Fig. 4:**
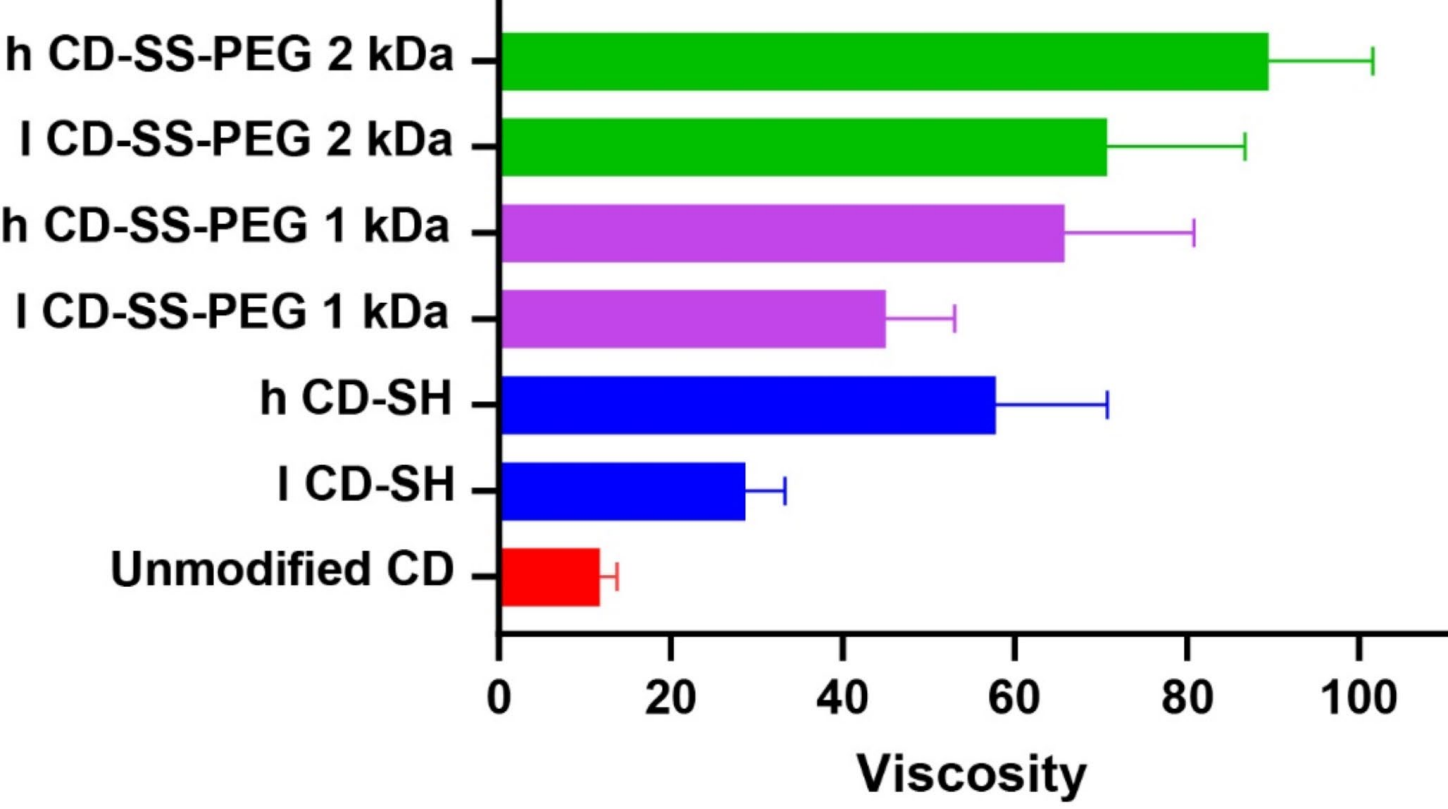
Dynamic viscosity of 0.5% (m/v) unmodified, thiolated, and S-protected thiolated CD incubated with mucus in a weight ratio of 1:5 at 37 °C for 3 h. Samples were prepared in 0.1 M phosphate buffer pH 6.8. Indicated values are means ± standard deviation of at least three experiments

Thiolated CDs form disulfide bonds with cysteine-rich glycoproteins in mucus, leading to the creation of new disulfide cross-links that increase the viscosity of the mixture. This in-situ gelling property of thiolated CDs, particularly in the presence of proteins with thiol groups, has been confirmed by recent studies. Rheological analyses were performed on mixtures of unmodified, thiolated, and S-protected thiolated CDs with mucus at 37 °C. The findings are summarized in Fig. 4. The viscosity of the unmodified CD/mucus mixture remained relatively stable over 3 h, serving as the control. In contrast, H CD-SH, H CD-SS-PEG 1 K, and PEG H CD-SS-PEG 2 K increased viscosity 5.5-, 7-, and 9-fold within this time period, respectively. The CD-SS-PEG indicated a higher increase than the unmodified and CD-SH. These outcomes may result from the enhanced interactions between PEG-modified CDs of different chain lengths and the mucus, involving both covalent bonding and non-covalent forces within the polymer-mucus mixture. Furthermore, the delayed cleavage of the less reactive PEG ligand could contribute to this effect by allowing greater interpenetration and increased interaction between the polymer and mucus.

#### In vitro mucoadhesion studies

**Fig. 5:**
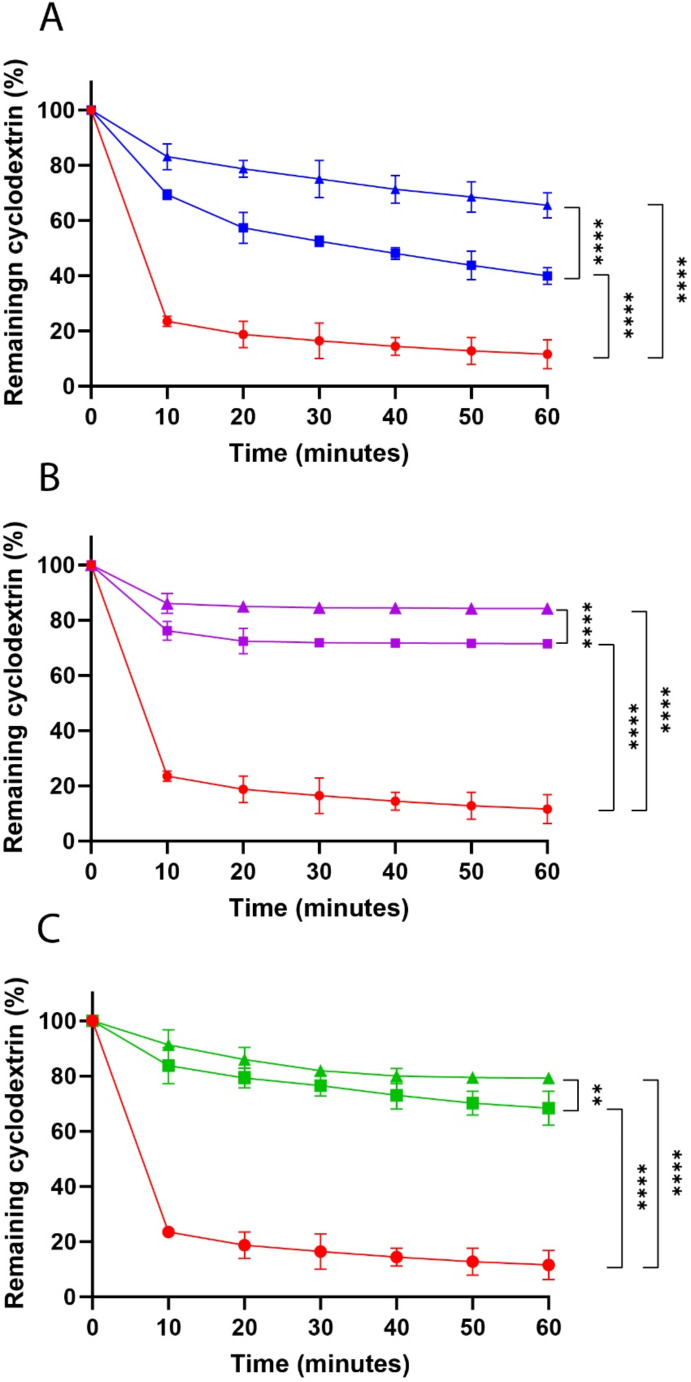
Percentage of remaining C6 labeled CD-SH (**A**), CD-SS-PEG 1 kDa (**B**), and CD-SS-PEG 2 kDa (**C**) on porcine intestinal mucosa under continuous rinsing with 0.1 M phosphate buffer pH 6.8 at 37° C and 100% relative humidity (● unmodified, ■ low, ▲ high degree of thiolation). Indicated values are means of at least three experiments ± standard deviation. Significant differences are indicated as ∗ ∗ ∗ *p* < 0.001; ∗ ∗ ∗ ∗ *p* < 0.0001

Thiolated CDs are believed to significantly enhance mucoadhesion, thereby extending their retention on mucosal surfaces. This prolonged residence time is particularly advantageous for drugs with limited absorption in the small intestine, as it can enhance systemic uptake. As illustrated in Fig. 5, after one hour, over 70% of h CD-SH, 85% of h CD-SS-PEG 1 kDa, and 80% of h CD-SS-PEG 2 kDa remained adhered to the intestinal mucosa, whereas unmodified CD was nearly completely washed away. This translates to a 7-, 8.4-, and 7.9-fold increase in mucoadhesion for h CD-SH, h CD-SS-PEG 1 kDa, and h CD-SS-PEG 2 kDa, respectively, compared to unmodified CD. In general, a higher degree of thiolation correlates with stronger mucoadhesive properties, a trend that was consistent across all thiolated CDs, regardless of the protective group used. Another factor contributing to the initially higher retention of 3rd generation thiolated CDs on the mucosa might be the lower reactivity of the ligand, which results in reduced binding to the loose mucins that are quickly washed away from the mucus layer. This allows more ligands to remain available for interaction with thiols in the deeper layers of mucus. These findings align with previous research from our group, suggesting that less reactive ligands may enhance interactions with mucosal tissues [18, 19].

### Cellular uptake studies

**Fig. 6:**
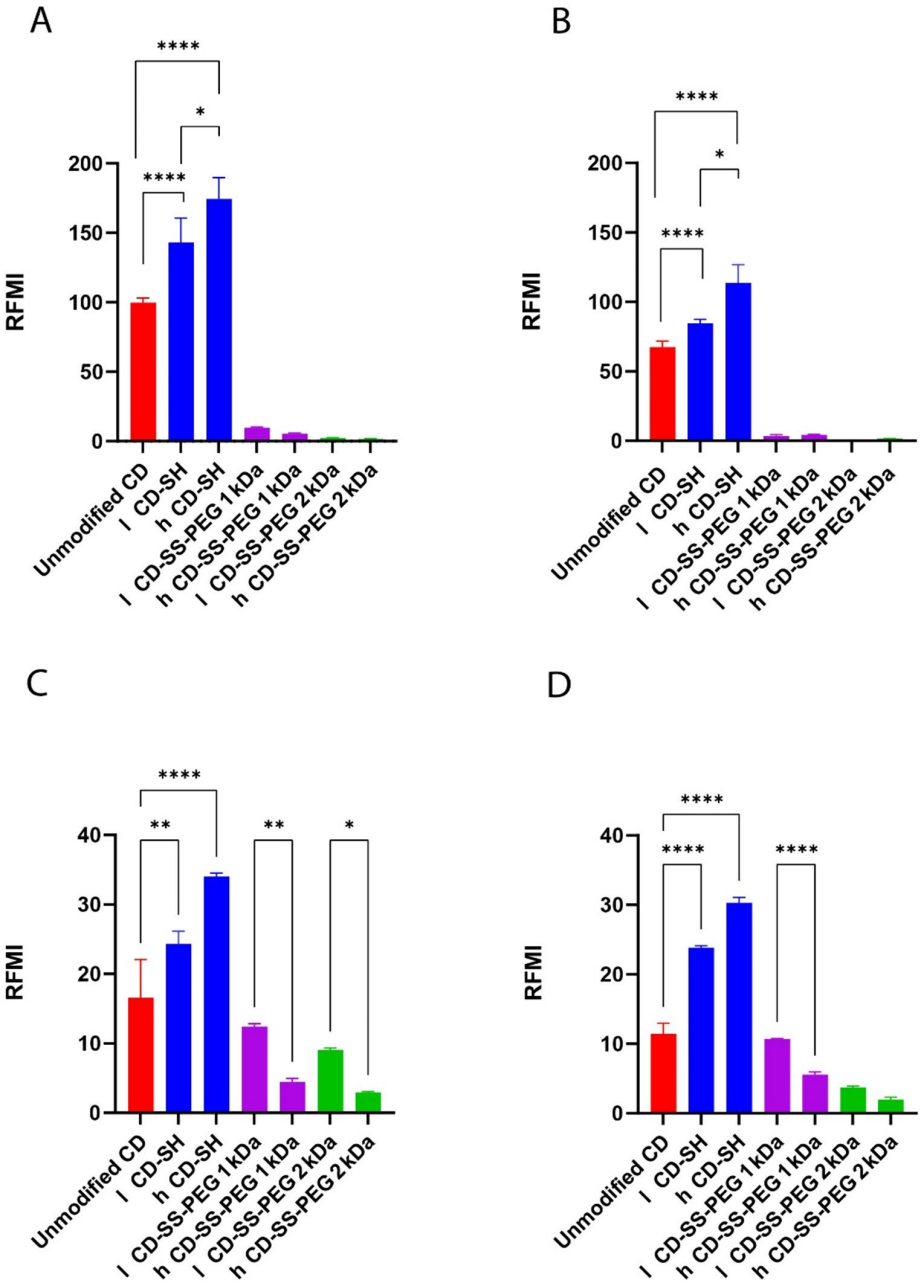
In vitro cellular uptake on Caco-2 cells at 37 °C (**A**), 4 °C (**B**), and cellular uptake on Hela cells at 37 °C (**C**) and 4 °C (**D**). Values are indicated as mean ± SD (*n* ≥ 3). **** *p* < 0.0001, *** *p* < 0.001, ** *p* < 0.01, * *p* < 0.01

To evaluate the cellular uptake of unmodified versus thiolated CDs, complexes were formed with the fluorescent hydrophobic dye C6. The results of the RMFI calculation are presented in Fig. 6. The observed increase in fluorescence intensity indicated that cells treated with CD-SH showed significantly higher uptake compared to those treated with unmodified CD or CD-SS-PEG. To gain deeper insight into the mechanisms behind the uptake of unmodified and thiolated β-CD, we conducted experiments at 4 °C to explore the potential involvement of an energy-dependent endocytosis pathway. Since most endocytic processes rely on energy, their activity is inhibited at lower temperatures due to reduced ATP consumption, effectively blocking active transport [20]. For the Caco-2 cell line, a significant reduction in cellular uptake was noted at 4 °C compared to 37 °C. This decline at lower temperatures suggests that the internalization of β-CDs occurs through an energy-dependent endocytosis pathway. Among all tested cell lines, CD-SH showed the highest uptake even at 4 °C. These findings indicate that thiolation markedly enhances the cellular uptake of β-CD, confirming that the uptake mechanism involves energy-dependent endocytosis.

### In vivo studies

**Fig. 7:**
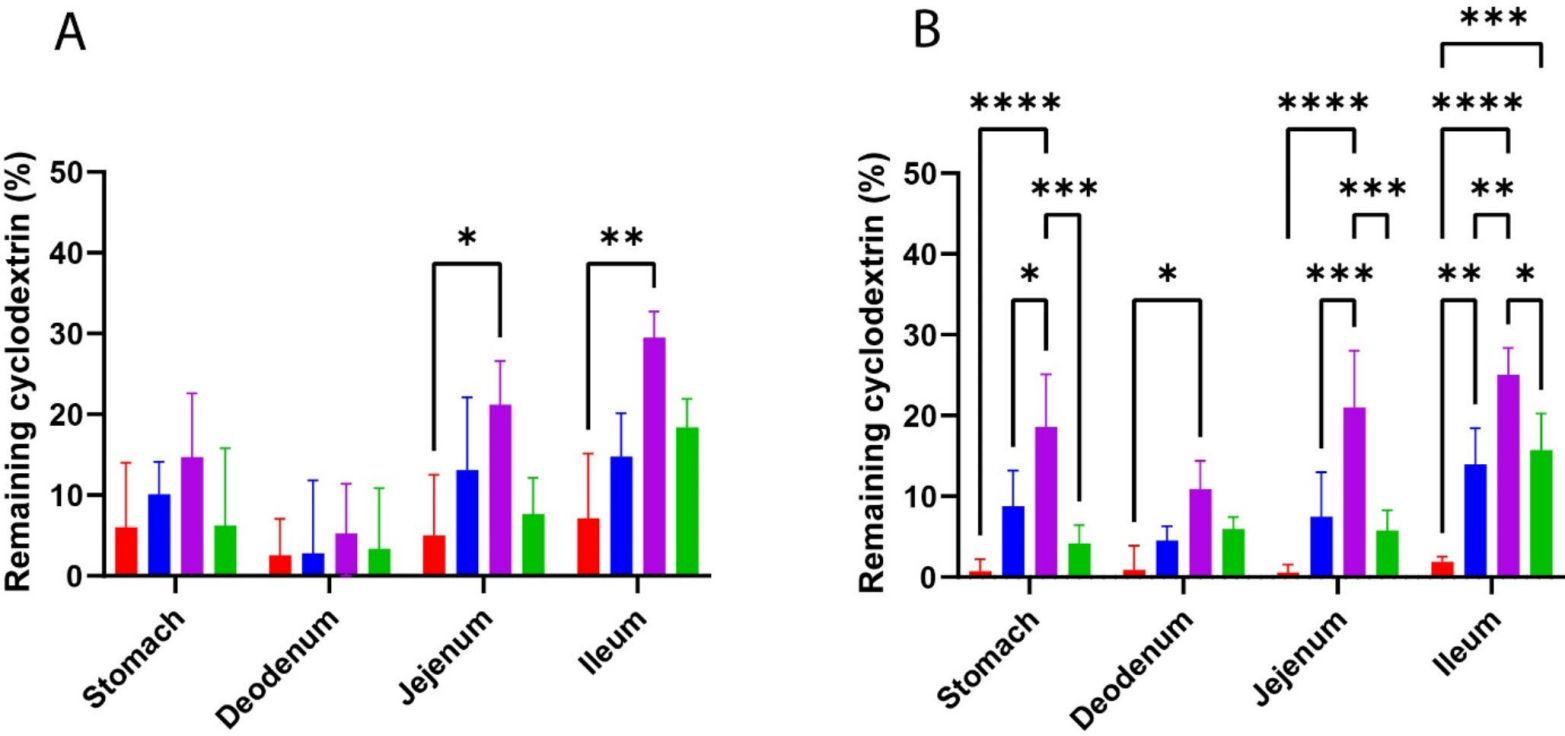
Graphical representation of the distribution of unmodified β-CD (red columns), h CD-SH (blue columns), h CD-SS-PEG 1 kDa (purple columns), and h CD-SS-PEG 2 kDa (green columns) in the gastrointestinal tract of male Sprague-Dawley rats after 4 (**A**) and 8 (**B**) hours. The data are shown as mean ± SD (*n* = 4) (***P < 0.001, *P > 0.01)

Building on the strong ex vivo mucoadhesive performance and minimal toxicity of thiolated CDs, an in vivo study was conducted to explore the mucoadhesive behavior of thiolated β-CD. The distribution patterns of C6-labeled unmodified, thiolated, and S-protected thiolated β-CDs were examined in Sprague-Dawley rats following oral administration, with results depicted in Fig. 7. After 4 h, approximately 35% of the dye complexed with CD-SS-PEG 1 kDa was found in the ileum, while 25% remained in the jejunum. In comparison, the unmodified CD showed only 8.5% retention in the ileum and 5% in the jejunum. Notably, CD-SS-PEG 1 kDa displayed a fourfold higher retention in the GI tract compared to unmodified CD after 4 h, likely due to its ability to penetrate deeper into the mucus layers, where the thiol groups are protected. Although the difference in retention was less pronounced in the duodenum, there was still a 1.5-fold increase following S-protection with 1 kDa PEG. After 8 h, the ileum contained 9.3 times more CD-SS-PEG 1 kDa than the unmodified CD/C6 complex. By this time, most of the unmodified CD complex had been cleared, whereas nearly 40% of the CD-SS-PEG 1 kDa/C6 complex remained in the GI tract, primarily in the ileum. These results highlight the effectiveness of S-protection in extending GI residence time. This approach could be particularly beneficial for treating GI diseases locally, as it may reduce dosing frequency and help maintain a consistent drug concentration in the bloodstream. The enhanced in vivo distribution of CD-SS-PEG 1 kDa is likely due to several factors, including the smaller PEG chains (1 kDa), which may offer superior conformability and penetration into mucosal layers compared to larger PEG chains (2 kDa) (Fig. 7).

## Discussion

The mucus layer in the gastrointestinal tract plays a key role in managing interactions within the intestinal lumen, including those involving orally delivered drugs, microbial communities, the epithelial and immune systems beneath it [21]. If systems are designed to be mucoadhesive, one question arises: How much of a given drug carrier adheres to and potentially diffuses through mucus as fluid transits through the GI tract? Medications taken orally offer distinct benefits compared to injectable, suppository, ocular, and nasal delivery methods, mainly due to their ease of use and improved patient compliance [22]. Despite extensive demand and emerging advantages, over 50% of therapeutic molecules are not available in oral form due to their physicochemical properties [23]. Thiolated CDs and other mucoadhesive delivery systems help prolong drug residence time in the GI tract, which can improve absorption and overall bioavailability [24]. Nevertheless, the task of securing these mucoadhesive systems in the ever-changing environment of the GI tract proves to be demanding, primarily due to the constant regeneration of the mucus layer. To surmount this challenge, mucoadhesive drug delivery systems must access and attach to the firm underlying mucus layer. S-protected thiolated CDs have the capability to merge strong mucoadhesive characteristics with the ability to penetrate the mucus layer [11]. This is achieved through cross-linking with the resilient mucus, thereby enhancing stability even further. By combining the benefits of both mucoadhesive and mucopenetrating systems, this approach effectively prolongs drug residence in the gastrointestinal tract. As illustrated in Fig. 4, the viscosity of mucus/CD combinations is impacted by the extent of thiolation and the specific S-protections employed. Thiolated CDs establish covalent connections through thiol-disulfide exchange reactions with the cysteine components of glycoproteins present in the mucus layer [25]. As the degree of thiolation increases, the viscosity of the mixture likewise escalates due to the augmented cross-linking density, consistent with findings from earlier investigations [26]. Due to the S-protection, a slight increase in viscosity was generally detected compared to the thiolated CDs. CD-SS-PEG 2 kDa, the third generation of thiolated CDs protected using PEG with a molar mass of 2 kDa, demonstrated the highest increase in mucus viscosity. This phenomenon can be attributed to diffusion of PEG S-protected thiolated CDs into deeper mucus layers. The selection of PEG chain length holds substantial influence over the characteristics of the resultant PEGylated nanocarrier, encompassing attributes such as its viscosity. As a result of the extended PEG chains, enhanced surface coverage occurs on the nanocarrier, thereby promoting steric impediment of particles [27]. This effect engenders augmented intermolecular interactions among PEG chains, consequently curtailing the motility of particles and imparting escalated viscosity. Moreover, the elevated density of PEG moieties at the nanocarrier interface can yield amplified steric repercussions and foster interactions among the PEG constituents, consequently increasing viscosity.

On the other hand, the third generation of thiolated CDs displayed a higher mucoadhesive improvement than CD-SH. The process of PEGylation introduces a stealth-like effect facilitated by the “brush-like” conformation of PEG chains [28]. This phenomenon acts as a deterrent against swift elimination by the mucus layer and the mucociliary clearance mechanism. The stealth effect extends the duration of stay of the nanocarrier on the mucosal surface, consequently permitting more efficacious interaction and adhesion. PEGylation shields thiol groups from untimely interactions and reactions [11]. This protective measure safeguards the potential of thiol groups to establish disulfide bonds with mucin upon reaching the deeper mucosal layer, thereby augmenting mucoadhesion. Thiolation furnishes specific binding sites that facilitate interaction with mucin, whereas PEGylation imparts heightened flexibility, hydration, and evasion of rapid clearance. Collectively, these attributes engender a nanocarrier that adheres more proficiently to the mucosal layer.

Additionally, higher cellular uptake in case of CD-SH can be attributed to thiol-mediated cellular uptake mechanisms such as endocytosis, fusion, and direct translocation across the plasma membrane to the cytosol [29]. By introducing thiol groups, the carrier system improves drug transport into cells through interactions with surface thiol residues, ultimately enhancing cellular uptake efficiency [30]. Thiol groups can interact with specific cell surface proteins, including CLIC1 chloride channels, EGFR, transferrin receptors, protein-disulfide isomerase, and TMEM16F scramblase, which have been recently explored as potential targets [31]. Studies suggest that modifying carrier systems with thiol groups improves their ability to penetrate cells more effectively than non-thiolated versions, primarily through thiol-disulfide exchange mechanisms [32–34]. However, the observed phenomenon of diminished cellular uptake upon introducing PEG, particularly in the context of heightened PEG chain density (2 kDa), can be ascribed to many factors that pertain to the interplay between CD-based nanocarriers and the cellular milieu. The process of cellular nanoparticle uptake is intricate and subject to a spectrum of influences, encompassing nanoparticle dimensions, surface characteristics, and engagements with cellular constituents [35]. PEGylation is recognized for conferring a “camouflage” effect on nanoparticles by establishing a hydrophilic exterior layer, thereby mitigating interactions with cellular proteins and components [36, 37]. This effect can impede the recognition of nanocarriers by cells, leading to decreased uptake [38, 39]. The supplementation of PEG, notably of higher molar mass (2 kDa), has the propensity to establish a more prominent steric barricade that restricts direct interchanges between nanocarriers and cell membranes [40]. The heightened surface density of PEG chains can yield a more robust steric hindrance, thereby impeding access to prospective cell surface receptors. A plethora of cellular uptake mechanisms entails receptor-mediated endocytosis, wherein the internalization process is facilitated by interactions between ligands on the nanocarrier surface and cell surface receptors [41]. Elevated PEG density might disrupt these interactions, culminating in diminished uptake. The length of PEG chains can also govern their associations with cell membranes. Longer PEG chains (2 kDa), in particular, could curtail the tendency of nanocarriers to engage with the cell membrane, thereby decelerating or impeding the uptake process.

Different formulations were tested for in vivo mucoadhesion studies, namely unmodified b-CD, highly thiolated β-CD, highly thiolated S-protected β-CD with PEG 1 kDa, and PEG 2 kDa. The incorporation of PEG 1 kDa has demonstrated a substantial prolongation in residence time, particularly in comparison to CD-SH, CD-SS-PEG 2 kDa, and the unmodified thiolated CD. The results, comparing the PEGs with different chain lengths, agree with the hypothesis of Huang et al., showing that increasing PEG chain length can lead to lower mucoadhesive properties [42]. They hypothesized that because higher PEG chains could form more self-entanglements than lower PEG chains, there would be less interpenetration of higher PEG chains into the mucin gel and, thus, less mucoadhesion [43, 44].

Furthermore, this observation can be elucidated by multiple factors about the molecular attributes and interactions inherent to the distinct compositions. PEG 1 kDa boasts a diminished molecular weight, consequently resulting in a more compact structure relative to PEG 2 kDa. The reduced dimensions of PEG 1 kDa enable the formation of a denser and more efficacious hydrophilic layer on the nanocarrier’s surface. This layer effectively mitigates engagements with mucins and other mucosal constituents, facilitating an elongated presence on the mucosal interface.

Thiolated CDs intrinsically possess mucoadhesive characteristics attributed to their capacity for disulfide bond formation with mucus glycoproteins. Combined with PEG 1 kDa, the nanocarriers presumably maintain a delicate equilibrium between mucoadhesion and steric hindrance. This equilibrium permits adherence to the mucosal surface while circumventing excessive interactions that could precipitate untimely clearance.

The lower molecular weight of PEG 1 kDa is anticipated to yield a more favorable surface density on the nanocarriers. This augmented density contributes to optimal coverage, thus amplifying mucoadhesive attributes while upholding desirable steric effects. PEGylation augments the nanocarriers’ capability to permeate the mucin layer, overlaying the mucosal surface. This heightened permeation facilitates a closer and more enduring interplay between the nanocarriers and the underlying mucosal tissues.

## Conclusion

Gastrointestinal mucus plays a crucial role in drug-carrier interactions in the gut. Mucoadhesive drug delivery systems, like S-protected thiolated CDs, are designed to enhance drug bioavailability in the GI tract. S-protection via PEGylation, especially with 1 kDa PEG chains, improves mucoadhesion by creating a hydrated envelope around nanocarriers, prolonging their presence in the mucosal gel layer. However, due to steric effects, longer PEG chain densities, such as 2 kDa, might hinder cellular uptake. In vivo studies have confirmed that PEG 1 kDa extends nanocarrier residence time. The combined approach of thiolation and PEGylation holds promise for improving oral drug delivery, bridging gaps in therapeutic molecule properties. Further research can unlock new opportunities in this field.

## Electronic supplementary material

Below is the link to the electronic supplementary material.

Supplementary Material 1

Supplementary Material 2

## Data Availability

The authors commit to providing data and materials supporting the findings and analyses in this paper upon reasonable request.

## References

[1] Kali G, Haddadzadegan S, Bernkop-Schnürch A. Cyclodextrins and derivatives in drug delivery: new developments, relevant clinical trials, and advanced products. Carbohydr Polym (2023) 121500. 10.1016/j.carbpol.2023.121500 37985088

[2] Puskás I, Szente L, Szőcs L, Fenyvesi É. Recent list of cyclodextrin-containing drug products. Periodica Polytech Chem Eng. 2023;67:11–7.

[3] Tiwari G, Tiwari R, Rai AK. Cyclodextrins in delivery systems: applications. J Pharm Bioallied Sci. 2010;2:72. 21814436 10.4103/0975-7406.67003 PMC3147107

[4] Kali G, Haddadzadegan S, Laffleur F, Bernkop-Schnürch A. Per-thiolated cyclodextrins: nanosized drug carriers providing a prolonged Gastrointestinal residence time. Carbohydr Polym. 2023;300:120275. 36372469 10.1016/j.carbpol.2022.120275

[5] Asim MH, Ijaz M, Rösch AC. Bernkop-Schnürch, thiolated cyclodextrins: new perspectives for old excipients. Coord Chem Rev. 2020;420:213433.

[6] Ricci F, Racaniello GF, Lopedota A, Laquintana V, Arduino I, Lopalco A, Cutrignelli A, Franco M, Sigurdsson HH, Denora N. Chitosan/sulfobutylether-β-cyclodextrin based nanoparticles coated with thiolated hyaluronic acid for indomethacin ophthalmic delivery. Int J Pharm. 2022;622:121905. 35697201 10.1016/j.ijpharm.2022.121905

[7] Bonengel S, Bernkop-Schnürch A. Thiomers—from bench to market. J Controlled Release. 2014;195:120–9. 10.1016/j.jconrel.2014.06.047 24993428

[8] Asim MH, Ijaz M, Mahmood A, Knoll P, Jalil A, Arshad S. Bernkop-Schnürch, thiolated cyclodextrins: mucoadhesive and permeation enhancing excipients for ocular drug delivery. Int J Pharm. 2021;599:120451. 33675922 10.1016/j.ijpharm.2021.120451

[9] Veider F, Haddadzadegan S, Armengol ES, Laffleur F, Kali G. Bernkop-Schnürch, Inhibition of P-glycoprotein-mediated efflux by thiolated cyclodextrins. Carbohydr Polym. 2024;327:121648. 38171673 10.1016/j.carbpol.2023.121648

[10] Fürst A, Kali G, Efiana NA, Akkuş-Dağdeviren ZB, Haddadzadegan S. Bernkop-Schnürch, thiolated cyclodextrins: A comparative study of their mucoadhesive properties. Int J Pharm. 2023;635:122719. 36791998 10.1016/j.ijpharm.2023.122719

[11] Haddadzadegan S, Knoll P, Wibel R, Kali G, Bernkop-Schnürch A. Three generations of thiolated cyclodextrins: A direct comparison of their mucus permeating and mucoadhesive properties. Acta Biomater. 2023;167:309–20. 37271247 10.1016/j.actbio.2023.05.050

[12] Leichner C, Jelkmann M, Bernkop-Schnürch A. Thiolated polymers: bioinspired polymers utilizing one of the most important bridging structures in nature. Adv Drug Deliv Rev. 2019;151:191–221. 31028759 10.1016/j.addr.2019.04.007

[13] Asim MH, Jalil A, Shahzadi I, Khan M, Matuszczak B, Bernkop-Schnürch A. Mucoadhesive S-protected thiolated cyclodextrin-iodine complexes: a promising strategy to prolong mucosal residence time of iodine. Future Microbiol. 2019;14:411–24. 30854897 10.2217/fmb-2018-0288

[14] Netsomboon K, Jalil A, Laffleur F, Hupfauf A, Gust R. Bernkop-Schnürch, thiolated chitosans: are Cys-Cys ligands key to the next generation? Carbohydr Polym. 2020;242:116395. 32564864 10.1016/j.carbpol.2020.116395

[15] Kali G, Knoll P, Bernkop-Schnürch A. Emerging technologies to increase Gastrointestinal transit times of drug delivery systems. J Controlled Release. 2022;346:289–99. 10.1016/j.jconrel.2022.04.016 35461970

[16] To D, Blanco Massani M, Coraça-Huber DC, Seybold A, Ricci F, Zöller K. A. Bernkop-Schnürch, Antibiotic-Polyphosphate nanocomplexes: A promising system for effective biofilm eradication. Int J Nanomed (2024) 9707–25. 10.2147/IJN.S473241 PMC11416784 39309185

[17] Jörgensen AM, Knoll P, Haddadzadegan S, Fabian H, Hupfauf A, Gust R, Jörgensen RG. Bernkop-Schnürch, biodegradable arginine based steroid-surfactants: cationic green agents for hydrophobic ion-pairing. Int J Pharm. 2023;630:122438. 36464112 10.1016/j.ijpharm.2022.122438

[18] Shahzadi I, Fürst A, Akkus-Dagdeviren ZB, Arshad S, Kurpiers M, Matuszczak B. Bernkop-Schnürch, less reactive thiol ligands: key towards highly mucoadhesive drug delivery systems. Polym (Basel). 2020;12:1259. 10.3390/polym12061259 PMC7362194 32486313

[19] Knoll P, Le N-MN, Wibel R, Baus RA, Kali G, Asim MH. Bernkop-Schnürch, thiolated pectins: in vitro and ex vivo evaluation of three generations of thiomers. Acta Biomater. 2021;135:139–49. 34418540 10.1016/j.actbio.2021.08.016

[20] Hsu Y-H, Hsieh H-L, Viswanathan G, Voon SH, Kue CS, Saw WS, Yeong CH, Azlan CA, Imae T, Kiew LV. Multifunctional carbon-coated magnetic sensing graphene oxide-cyclodextrin nanohybrid for potential cancer theranosis. J Nanopart Res. 2017;19:1–19.

[21] Wang C-M, Fernez MT, Woolston BM, Carrier RL. Native Gastrointestinal mucus: models and techniques for studying interactions with drugs, drug carriers, and Bacteria. Adv Drug Deliv Rev (2023) 114966. 10.1016/j.addr.2023.114966 PMC11184232 37329985

[22] Haddadzadegan S, Dorkoosh F, Bernkop-Schnürch A. Oral delivery of therapeutic peptides and proteins: technology landscape of lipid-based nanocarriers. Adv Drug Deliv Rev. 2022;182:114097. 34999121 10.1016/j.addr.2021.114097

[23] Kumar R, Islam T, Nurunnabi M. Mucoadhesive carriers for oral drug delivery. J Controlled Release. 2022;351:504–59. 10.1016/j.jconrel.2022.09.024 PMC9960552 36116580

[24] Li J, Wang X, Zhang T, Wang C, Huang Z, Luo X, Deng Y. A review on phospholipids and their main applications in drug delivery systems. Asian J Pharm Sci. 2015;10:81–98.

[25] Mfoafo K, Mittal R, Eshraghi A, Omidi Y, Omidian H. Thiolated polymers: an overview of mucoadhesive properties and their potential in drug delivery via mucosal tissues. J Drug Deliv Sci Technol (2023) 104596.

[26] Jalil A, Asim MH, Le N-MN, Laffleur F, Matuszczak B, Tribus M. Bernkop–Schnürch, S-protected Gellan gum: decisive approach towards mucoadhesive antimicrobial vaginal films. Int J Biol Macromol. 2019;130:148–57. 30779984 10.1016/j.ijbiomac.2019.02.092

[27] Su G, Jiang H, Xu B, Yu Y, Chen X. Effects of protein Corona on active and passive targeting of Cyclic RGD peptide-functionalized pegylation nanoparticles. Mol Pharm. 2018;15:5019–30. 30222356 10.1021/acs.molpharmaceut.8b00612

[28] Suk JS, Xu Q, Kim N, Hanes J, Ensign LM. PEGylation as a strategy for improving nanoparticle-based drug and gene delivery. Adv Drug Deliv Rev. 2016;99:28–51. 26456916 10.1016/j.addr.2015.09.012 PMC4798869

[29] Kali G, Taha AMMM, Campanella E, Truszkowska M, Haddadzadegan S, Denora N. A. Bernkop-Schnürch, enhanced mucoadhesion of thiolated β-Cyclodextrin by S-Protection with 2-Mercaptoethanesulfonic acid. ACS Omega (2024). 10.1021/acsomega.3c08836 PMC10851230 38343993

[30] Kaplan Ö, Truszkowska M, Kali G, Knoll P, Massani MB, Braun DE. A. Bernkop-Schnürch, thiolated α-cyclodextrin: the likely smallest drug carrier providing enhanced cellular uptake and endosomal escape. Carbohydr Polym (2023) 121070. 10.1016/j.carbpol.2023.121070 37321712

[31] Laurent Q, Martinent R, Lim B, Pham A-T, Kato T, López-Andarias J, Sakai N, Matile S. Thiol-mediated uptake. JACS Au. 2021;1:710–28. 34467328 10.1021/jacsau.1c00128 PMC8395643

[32] Zhang R, Qin X, Kong F, Chen P, Pan G. Improving cellular uptake of therapeutic entities through interaction with components of cell membrane. Drug Deliv. 2019;26:328–42. 30905189 10.1080/10717544.2019.1582730 PMC6442206

[33] Knoll P, Racaniello GF, Laquintana V, Veider F, Saleh A, Seybold A, Denora N. Bernkop-Schnürch, Lipid-based nanoparticles: enhanced cellular uptake via surface thiolation. Int J Pharm. 2023;635:122753. 36863545 10.1016/j.ijpharm.2023.122753

[34] Li T, Takeoka S. Enhanced cellular uptake of maleimide-modified liposomes via thiol-mediated transport. Int J Nanomed (2014) 2849–61. 10.2147/IJN.S58540 PMC4051732 24940060

[35] Behzadi S, Serpooshan V, Tao W, Hamaly MA, Alkawareek MY, Dreaden EC, Brown D, Alkilany AM, Farokhzad OC, Mahmoudi M. Cellular uptake of nanoparticles: journey inside the cell. Chem Soc Rev. 2017;46:4218–44. 28585944 10.1039/c6cs00636a PMC5593313

[36] Mishra S, Webster P, Davis ME. PEGylation significantly affects cellular uptake and intracellular trafficking of non-viral gene delivery particles. Eur J Cell Biol. 2004;83:97–111. 15202568 10.1078/0171-9335-00363

[37] Barrios-Gumiel A, Sanchez-Nieves J, Pedziwiatr-Werbicka E, Abashkin V, Shcharbina N, Shcharbin D, Glińska S, Ciepluch K, Kuc-Ciepluch D, Lach D. Effect of pegylation on the biological properties of cationic carbosilane dendronized gold nanoparticles. Int J Pharm. 2020;573:118867. 31765788 10.1016/j.ijpharm.2019.118867

[38] Fishburn CS. The Pharmacology of pegylation: balancing PD with PK to generate novel therapeutics. J Pharm Sci. 2008;97:4167–83. 18200508 10.1002/jps.21278

[39] Zhang X, Wang H, Ma Z, Wu B. Effects of pharmaceutical pegylation on drug metabolism and its clinical concerns. Expert Opin Drug Metab Toxicol. 2014;10:1691–702. 25270687 10.1517/17425255.2014.967679

[40] Lee H, Larson RG. Adsorption of plasma proteins onto pegylated lipid bilayers: the effect of PEG size and grafting density. Biomacromolecules. 2016;17:1757–65. 27046506 10.1021/acs.biomac.6b00146

[41] Mazumdar S, Chitkara D, Mittal A. Exploration and insights into the cellular internalization and intracellular fate of amphiphilic polymeric nanocarriers. Acta Pharm Sin B. 2021;11:903–24. 33996406 10.1016/j.apsb.2021.02.019 PMC8105776

[42] Huang Y, Leobandung W, Foss A, Peppas NA. Molecular aspects of muco-and bioadhesion:: tethered structures and site-specific surfaces. J Controlled Release. 2000;65:63–71. 10.1016/s0168-3659(99)00233-3 10699271

[43] Maisel K, Reddy M, Xu Q, Chattopadhyay S, Cone R, Ensign LM, Hanes J. Nanoparticles coated with high molecular weight PEG penetrate mucus and provide uniform vaginal and colorectal distribution in vivo. Nanomedicine. 2016;11:1337–43. 27171816 10.2217/nnm-2016-0047 PMC4897967

[44] De Ascentiis A, Degrazia JL, Bowman CN, Colombo P, Peppas NA. Mucoadhesion of Poly (2-hydroxyethyl methacrylate) is improved when linear Poly (ethylene oxide) chains are added to the Polymer network. J Controlled Release. 1995;33:197–201.

