# Supplementary material for: Cyclodextrin-mediated enhancement of gastrointestinal drug delivery: unveiling mucoadhesive and mucopenetrating synergy
Source: Drug Deliv Transl Res. 2025 Mar 20;15(10):3753–67. doi: 10.1007/s13346-025-01832-w (PMC12397179; doi:10.1007/s13346-025-01832-w)
Supplement: Supplementary file 1 — Supplementary Material 1 [file 13346_2025_1832_MOESM1_ESM.docx]

**
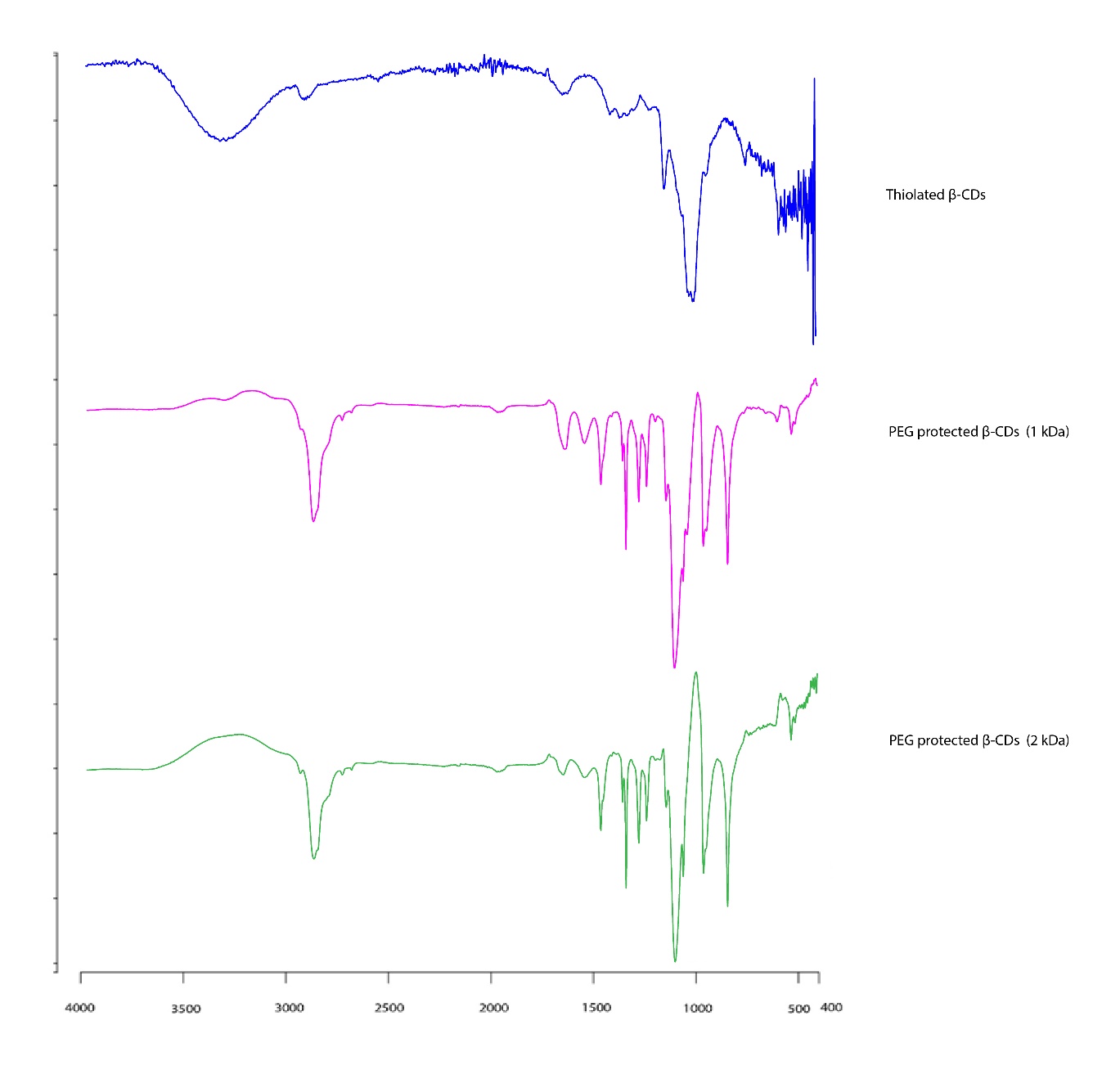
**

**Figure S.1. FT-IR spectra of Thiolated and PEG protected cyclodextrins.**

**
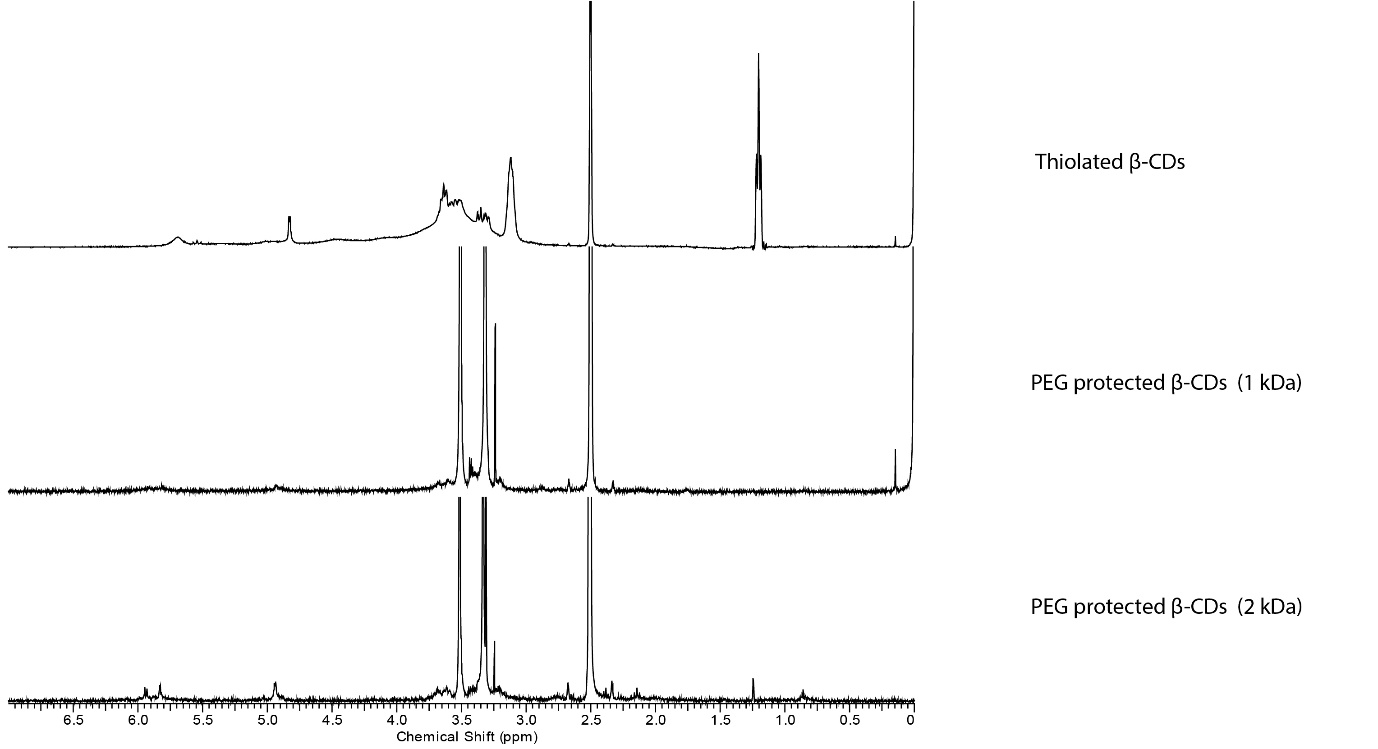
**

**Figure S.2. 400 MHz ^1^H NMR spectra of Thiolated and PEG protected cyclodextrins.**
